# Reliable reference genes for the quantification of mRNA in human T-cells and PBMCs stimulated with live influenza virus

**DOI:** 10.1186/s12865-020-0334-8

**Published:** 2020-01-31

**Authors:** Justin G. Roy, Janet E. McElhaney, Chris P. Verschoor

**Affiliations:** Health Sciences North Research Institute, 41 Ramsey Lake Rd, Sudbury, ON P3E5J1 Canada

**Keywords:** Influenza, Human peripheral blood mononuclear cells, T-cells, Reference genes, Housekeeping genes, Quantitative PCR

## Abstract

**Background:**

Quantitative PCR (qPCR) is a powerful tool that is particularly well-suited to measure mRNA levels in clinical samples, especially those with relatively low cell counts. However, a caveat of this approach is that reliable, stably expressed reference (housekeeping) genes are vital in order to ensure reproducibility and appropriate biological inference. In this study, we evaluated the expression stability of six reference genes in peripheral blood mononuclear cells (PBMCs) and isolated CD3^+^ T-cells from young and old adults (*n* = 10), following ex vivo stimulation with mock (unstimulated) or live influenza virus. Our genes included: β-actin (*ACTB*), glyercaldehyde-3-phostphate dehydrogenase (*GAPDH*), ribosomal protein L13a (*RPL13a*), ribosomal protein S18 (*RPS18*), succinate dehydrogenase complex flavoprotein subunit A (*SDHA*), and ubiquitin-conjugating enzyme E2D2 (*UBE2D2*).

**Results:**

Reference gene expression varied significantly depending on cell type and stimulation conditions, but not age. Using the comparative ΔCt method, and the previously published software BestKeeper, NormFinder, and geNorm, we show that in PBMCs and T-cells, *UBE2D2* and *RPS18* were the most stable reference genes, followed by *ACTB*; however, the expression of *UBE2D2* and *RPS18* was found to increase with viral stimulation in isolated T-cells, while *ACTB* expression did not change significantly. No age-related differences in stability were observed for any gene

**Conclusions:**

This study suggests the use of a combination of *UBE2D2*, *RPS18*, and *ACTB* for the study of influenza responses in PBMCs and T-cells, although *ACTB* alone may be the most optimal choice if choosing to compare target gene expression before and after viral stimulation. Both *GAPDH* and *RPL13a* were found to be poor reference genes and should be avoided for studies of this nature.

## Background

A powerful tool one can use to measure gene expression in immune cells, including human peripheral blood mononuclear cells (PBMCs) and T-cells, is quantitative polymerase chain reaction (qPCR). This technology provides high sensitivity when measuring mRNA [1], making it an ideal tool for gene expression analysis in fresh PBMCs and isolated T-cells. An important component of any well-designed qPCR study is the reference or housekeeping gene(s) employed, which must be chosen carefully and measured precisely [2, 3]. These genes are absolutely critical in order to control for differences in overall transcript abundance from sample to sample, and as such, have substantial influence on measured levels of target mRNAs [4, 5]. Therefore, before attempting to evaluate changes in gene expression within human PBMC and T-cell populations, the identification of stable reference genes must be performed under the conditions which they will be measured. Unfortunately, it has been demonstrated that no single gene can be used for all cell types and tissue types [6], and in some cases, multiple reference genes are required [7].

This study aimed to identify stable reference genes in human PBMCs and CD3^+^ T-cells for an assay commonly used to measure immunity following infection or vaccination to influenza A virus, a major respiratory pathogen [8, 9]. In addition to comparing reference gene stability with and without live influenza challenge ex vivo, we also compared stability between young and old adults, given that age is a major determinant of susceptibility to infection and can alter PBMC mRNA expression profiles significantly [10, 11]. Six candidate reference genes, chosen from the literature, were assessed in human PBMCs and T-cells. Commonly used genes such as *ACTB* and *GAPDH* were included even though many studies have demonstrated high variability in the expression of these genes under various conditions [2, 4, 12–14], including influenza infection [15]. *RPL13a* and *SDHA* have been found to be valid reference genes in both T-cells and mixed leukocytes [7], while *UBE2D2* was found to be the most stably expressed reference gene in different PBMC subsets of Multiple Sclerosis patients [16]. Finally, *RPS18* was included as it has been shown to be fairly stable in PBMCs from other species [17] and in tumour neovascularization studies [18]. We compared these genes using four methods, each of which estimating stability and/or reliability in a slightly differ manner: geNorm [7] determines gene expression stability (ie. M) by calculating the average pairwise variation of each reference gene; NormFinder [19] uses an ANOVA based approached to calculate the candidate gene stability value by estimating the expression variation within the overall group (intragroup) and between groups (intergroup); Bestkeeper [20] estimates reliability according to the standard deviation of Cq values and the Pearson correlation between a given gene and an index of the most stable reference genes, as determined by the software; Lastly, the comparative ΔCt method, proposed by Silver and colleagues [21], compares the relative expression of pairs of reference genes within the sample and uses the average standard deviation of the ΔCt (or ΔCq) for each reference gene as a measure of stability.

## Results

### Evaluation of candidate reference gene expression in unstimulated and influenza a stimulated PBMCs and T-cells

**Fig. 1:**
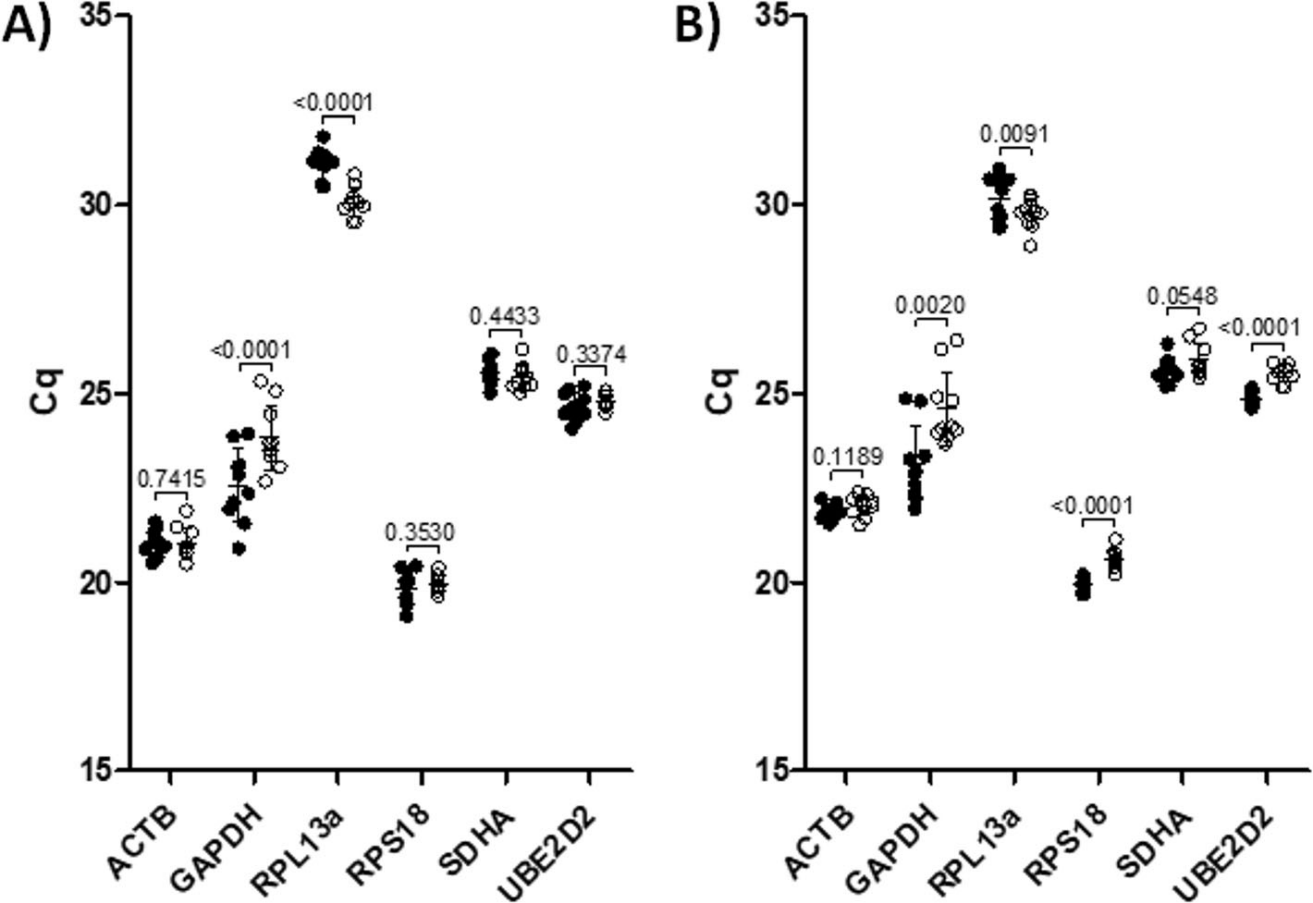
Cq values for candidate reference genes in stimulated and unstimulated PBMCs and T-cells. Mean quantification cycle (Cq) values are presented for both unstimulated and influenza A/Victoria/375 stimulated donor (*n* = 10) PBMCs (**a**) and CD3^+^ T-cells (**b**). Unstimulated controls are presented by the black, filled circles, and the paired stimulated samples are presented by clear circles. Statistical analysis was performed by paired t-test or Wilcoxon signed rank test. *P*-values are presented between indicated comparisons. Data from 10 young and old donors is presented

Using qPCR, the expression of each of the six candidate reference genes (Tables 1 and 2) was measured in PBMCs and T-cells from a combination of young and old donors (*n* = 10), in the presence or absence of influenza stimulation. When results from both treatments and cell types were combined, *RPS18* (mean Cq ~ 20) demonstrated the highest expression, followed by *ACTB* (~ 21.5)*, GAPDH* (~ 23.5)*, UBE2D2* (~ 25)*, SDHA* (~ 25.5) and *RPL13a* (~ 30.5); no significant difference between cell types were observed. In PBMC samples, both *GAPDH* (*p* < 0.001) and *RPL13a* (*p* < 0.001) were significantly different between unstimulated and stimulated treatments; the remaining candidate reference genes showed no significant differences (Fig. 1A). In isolated T-cells, four of six candidate genes were significantly different between treatments: *GAPDH* (*p* < 0.01)*, RPL13a* (*p* < 0.01)*, RPS18* (*p* < 0.001)*,* and *UBE2D2* (*p* < 0.001) (Fig. 1B). Age did not appear to have a significant effect on the expression of the candidate reference genes (Additional file 1: Fig. S1).

**Table 1:** Candidate reference genes selected for this study

Symbol	Name	Accession Number	Amplicon Size (bp)	IDT Assay ID
*ACTB*	β-actin	NM_001101.5	110	Hs.PT.39a.22214847
*GAPDH*	Glyercaldehyde-3-phostphate dehydrogenase	NM_002046.7	143	Hs.PT.39a.22214836
*RPL13a*	Ribosomal protein, L13a	NM_012423.4	106	Hs.PT.58.45725862
*RPS18*	Ribosomal protein, S18	NM_022551.3	130	Hs.PT.58.14390640
*SDHA*	Succinate dehydrogenase complex flavoprotein subunit A	NM_004168.4	150	Hs.PT.58.41017719
*UBE2D2*	Ubiquitin-conjugating enzyme E2D2	NM_181838.1	119	Hs.PT.58.622887

**Table 2:** Candidate reference gene primers and details

Symbol	Primer and Probe Sequences (5′-3′)	Exons	Average Efficiency (%)^a^
*ACTB*	F: ACAGAGCCTCGCCTTTG R: CCTTGCACATGCCGGAG P: /5HEX/TCATCCATG/ZEN/GTGAGCTGGCGG/3IABkFQ/	1–2	99.5
*GAPDH*	F: ACATCGCTCAGACACCATG R: TGTAGTTGAGGTCAATGAAGGG P: /5HEX/AAGGTCGGA/ZEN/GTCAACGGATTTGGTC/3AIBkFQ/	2–3	96.8
*RPL13a*	F: GCCGCCCCTGTTTCAAG R: CTCGACCATCAAGCACCAG P: /5HEX/AGAAACCCT/ZEN/GCGACAAAACCTCCT/3IABkFQ/	1b-3	99.3
*RPS18*	F: GTTCCAGCATATTTTGCGAGT R: GTCAATGTCTGCTTTCCTCAAC P: /5HEX/TCTTCGGCC/ZEN/CACACCCTTAATGG/3IABkFQ/	2–3	99.5
*SDHA*	F: TTTGATGCAGTGGTGGTAGG R: CAGAGCAGCATTGATTCCTC P: /5HEX/TGCAACAGT/ZEN/GTGTGACCTGGTAGG/3IABkFQ/	3–4	99.5
*UBE2D2*	F: GTACTCTTGTCCATCTGTTCTCTG R: CCATTCCCGAGCTATTCTGTT P: /5HEX/CCGAGCAAT/ZEN/CTCAGGCACTAAAGGA/3IABkFQ/	6–8	99.3

^a^ Average efficiencies were calculated from both unstimulated PBMC and T-cell samples

### Analysis of candidate reference genes using Silver’s method, geNorm, BestKeeper and NormFinder

Candidate reference gene expression was further analyzed through the use of previously published software and techniques (Table 3). With exception to the NormFinder approach, all analyses were performed on data pooled from young and old adults, and with and without virus stimulation.

**Table 3:** Reference gene reliability analysis on unstimulated and live influenza A virus stimulated PBMCs using Silver’s method, geNorm, BestKeeper and NormFinder

		*ACTB*		*GAPDH*		*RPL13a*		*RPS18*		*SDHA*		*UBE2D2*	
Method	Measure	*PBMC*	*T-cell*	*PBMC*	*T-cell*	*PBMC*	*T-cell*	*PBMC*	*T-cell*	*PBMC*	*T-cell*	*PBMC*	*T-cell*
Silver	σCq	0.52	0.562	1.204	1.29	0.803	0.821	0.538	0.568	0.564	0.601	0.493	0.541
geNorm	M	1.281	0.362	0.825	0.854	0.556	0.312	0.52	0.278	0.53	0.311	0.537	0.287
BestKeeper	SD	0.29	0.20	0.84	0.96	0.52	0.43	0.25	0.33	0.26	0.29	0.22	0.35
	r	0.737	0.504	0.587	0.688	0.227	−0.173	0.738	0.873	0.401	0.468	0.836	0.907
NormFinder (Stability Value)	Intragroup (all samples)	0.067	0.100	0.810	0.873	0.479	0.496	0.068	0.074	0.192	0.219	0.073	0.074
	Intergroup (± virus)	0.094	0.147	0.572	0.508	0.436	0.402	0.074	0.08	0.152	0.173	0.046	0.099
	Intergroup (Y/O)	0.034	0.039	0.26	0.281	0.154	0.161	0.044	0.029	0.058	0.066	0.022	0.023

Expression stability of candidate reference genes was calculated from *n* = 10 paired unstimulated and influenza A/Victoria/375 stimulated PBMCs and T-cells

With respect to Silver’s method, *UBE2D2* was determined to have the greatest stability in PBMCs (0.493), followed by *ACTB* (0.520), *RPS18* (0.538) and *SDHA* (0.564); this was similarly observed in isolated T-cells. For both PBMCs and T-cells, *GAPDH* was ranked as the least stable gene (1.204 and 1.290, respectively).

According to geNorm, all reference genes considered were deemed stable in both PBMCs and T-cells (M < 1.5, according to [7]); for both cell types, *RPS18* (PBMCs = 0.52, T-cells = 0.278), *SDHA* (0.53, 0.311) and *UBE2D2* (0.537, 0.287) were ranked the highest. Interestingly, *ACTB*, which was deemed suitable using Silver’s method in PBMCs, had the lowest expression stability value of the reference genes tested (1.28). In T-cells, *GAPDH* was deemed to be the least stable, however, still within the stability cut-off (0.854).

Using BestKeeper, all reference genes were determined to be relatively stable in both PBMCs and T-cells (SD < 1), according to [20]). In PBMCs, *UBE2D2* and *RPS18* were the most stable (ie. lowest SD), while exhibiting the highest correlations (*UBE2D2*: SD = 0.22, *r* = 0.836; *RPS18*: SD = 0.25, *r* = 0.738), followed by *ACTB* (SD = 0.29, *r* = 0.737); *SDHA* exhibited good stability (SD = 0.26), but a relatively low correlation (*r* = 0.401). In T-cells, *RPS18* and *UBE2D2* demonstrated the best stability and correlation (*RPS18*: SD = 0.33, *r* = 0.873; UBE2D2: SD = 0.35, *r* = 0.907), while *ACTB* and *SDHA* only exhibited good stability (0.20 and 0.29, respectively). *GAPDH* exhibited the worst stability across cell types (PBMCs = 0.84, T-cells = 0.96), while *RPL13a* exhibited the worst correlation (PBMCs = 0.227, T-cells = − 0.173).

We performed an intragroup analysis of all unstimulated and stimulated samples using NormFinder and found that *RPS18, UBE2D2* and *ACTB* were determined to have the lowest stability value (ie. best stability) in PBMCs and T-cells (Stability Value ≤0.10); this was similarly observed for the intergroup analyses, where stratification by treatment or age was considered. Although not deemed as stable as *RPS18, UBE2D2* and *ACTB*, the Stability Values observed for *SDHA* are notable (≤0.219).

### Scoring the best and worst reference genes according to stability

For our assessment of reference gene stability, we considered four different approaches and seven measures in total. To summarize these findings and conclude on the best and worst genes evaluated, we ranked each gene and assigned a score of 3, 2 or 1 if the gene was found to rank 1st, 2nd or 3rd best, respectively, for a given measure. For PBMCs, *UBE2D2* (score = 17) was best, followed by *RPS18* (13), *ACTB* (9), *SDHA* (3), and *GAPDH*/*RPL13a* (0). For T-cells, *UBE2D2* and *RPS18* scored similarly (15), followed by *ACTB* (8), *SDHA* (3), *GAPDH* (1) and *RPL13a* (0).

## Discussion

In this study, we evaluated the expression stability and suitability of candidate reference genes in influenza virus stimulated PBMCs and T-cells. Our data shows that *UBE2D2* and *RPS18* ranked the highest with regards to stability in both PBMCs and T-cells, followed closely by *ACTB*. However, both *UBE2D2* and *RPS18* were expressed significantly higher following viral stimulation in T-cells, but not *ACTB*. Whether considering overall stability or comparing expression with or without viral stimulation, *GAPDH* and *RPL13a* were ranked the worst in both cell types.

The software geNorm, NormFinder and Bestkeeper, and Silver’s method all provided similar results for both the PBMC and T-cell reference gene analysis, ranking *UBE2D2* as the most stable gene, followed by *RPS18*; this has been previously demonstrated [16, 18]. Furthermore, in PBMCs these genes were found to not be affected by age or viral stimulation. This is particularly important in the context of influenza, given the prominent role of age-related immune dysfunction in determining the susceptibility to infection or response to vaccination [22, 23]. Interestingly, the next highest ranked gene we identified, *ACTB*, has previously been demonstrated to have high variance in expression [14], including in T-cells [3, 24]. While this should suggest caution prior to implementing *ACTB* as a reference gene, it is worth noting that it was also one of the few genes that was not significantly different in virus stimulated and unstimulated cultures in PBMCs or T-cells. Hence, *ACTB* may warrant consideration for mRNA studies that are specifically comparing target gene expression before and after immune stimulation, especially since both *UBE2D2* and *RPS18* were significantly different with viral stimulation in isolated T-cells. As a whole, this demonstrates the importance of testing each specific experimental condition on potential reference genes as well as in specific sample types, prior to the measurement of target genes.

Our study has a number of strengths resulting in a comprehensive analysis that will help researchers decide on the best reference gene to use depending on their experimental conditions and study design. We compared six well-known reference genes using four different approaches and seven measures in total, and also stratified our analysis by cell type (PBMCs and isolated T-cells), age group (young and old), and experimental treatment (with and without virus). However, we concede that experimental conditions beyond that tested in our study may impact the stability of reference genes differently. Furthermore, it is possible that we were underpowered to investigate the effect of age on reference gene stability, given that others have shown there to be a notable effect, albeit in tissues [25, 26].

## Conclusions

The current Minimum Information for Publication of Quantitative Real-Time PCR Experiments (MIQE) suggests the use of more than one reference genes in all qPCR studies [4]. Hence, we recommend the use of *UBE2D2* and *RPS18*, or *UBE2D2*, *RPS18* and *ACTB* in studies of PBMCs or isolated T-cells. However, when comparing target gene expression across stimulations, we recommend the use of *ACTB* alone if studying isolated T-cells. In our analyses neither *GAPDH* or *RPL13a* were found to be reliable reference genes, and should be avoided unless properly evaluated under the intended research conditions.

## Methods

### Study design and participants

Frozen PBMC samples from a previous study [27] were used for the identification of reference genes. Ten samples, derived from heparinized blood collected 4-weeks following influenza vaccination, were chosen and consisted of five older donors (O; mean age [range] = 77 [70–80]) and five younger donors (Y; 30 [26–35]), and similarly balanced according to cytomegalovirus serostatus (O = 40% seropositive, Y = 60% seropositive).

### PBMC cell culture, stimulation and CD3^+^ T-cell isolation

To measure candidate reference gene expression, PBMCs were stimulated with live influenza virus, as previously described with some changes [28]. Briefly, frozen PBMC samples [27] were thawed, counted and adjusted to a concentration of 1 × 10^6^ cells/200 μL in Aim V media (Gibco) supplemented with Human AB Serum (Corning). PBMCs were placed in 96-well U-bottom plates (Corning) and were either left unstimulated or stimulated with live influenza A/Victoria/375 virus (sucrose-gradient purified, Charles River Laboratories) at a multiplicity of infection of six for 8 h at 37 °C, 5% CO_2_ in a humidified chamber. After incubation, cells for PBMC mRNA analysis were collected and stored in 350 μL RLT buffer (Qiagen) supplemented with 1% β-mercaptomethanol (Sigma-Aldrich) at − 80 °C. CD3^+^ T-cells were isolated from remaining cells using the EasySep Human T-cell Enrichment kit (Stem Cell Technologies), following manufacturer’s instructions and stored in RLT buffer at − 80 °C.

### RNA isolation and cDNA synthesis

RNA was isolated from PBMC and T-cell lysates using the RNeasy Mini Kit (Qiagen) according to manufacturer’s instructions. Isolated RNA was quantified using the NanoDrop 2000 Spectrophotometer (Thermo Scientific) and stored at − 80 °C; RNA with an 260/280 absorbance ratio below 1.75 were not tested further. Complementary DNA (cDNA) was synthesized from 100 ng of total RNA using of the High Capacity cDNA reverse transcription kit (Applied Biosystems) according to manufacturer’s recommendation (final volume = 20 μL), and stored at − 20 °C.

### Quantitative PCR and reference genes

Candidate reference gene primer/probe mixes were purchased from Integrated DNA Technologies as pre-validated probe PrimeTime® qPCR Probe Assays using TaqMan based chemistry (Table 1). Table 2 highlights the primer and probe sequences, location of the primers and the average efficiencies of the primers in previous experiments using 2-fold serial dilutions; primer efficiency was calculated as 10^(− 1/slope)^. Quantitative PCR was performed using the QuantStudio™ 5 Real Time PCR System (Applied Biosystems); briefly, 2 μL of cDNA of each sample was loaded in duplicate into 96-well plates and 8 μL of qPCR master mix was added, which included TaqMan Fast Advanced Master Mix (5 μL; Applied Biosystems), reference gene primer/probe mix (0.5 μL), and RNase/DNase free H_2_O (2.5 μL; Qiagen)). Negative controls (no RNA or cDNA) were included to verify the absence of contamination. Amplification was performed at 60 °C using a two-step cycling procedure for 40 cycles, and resultant quantification cycles (Cq) were calculated using the default settings in the QuantStudio Design & Analysis Software v1.4.3 (Applied Biosystems).

### Statistical analysis and identification of suitable reference genes

All statistical tests were performed using GraphPad Prism 5. Pairwise comparisons were performed by either the Wilcoxon signed ranked sum test or a Student’s paired t-test, depending on the normality of data, which was determined by the Shapiro-Wilks normality test. To evaluate reference gene stability, four separate methods were employed: geNorm [7], NormFinder [19], BestKeeper [20], and Silver’s ΔCt method [21]. For geNorm and NormFinder analyses, mean Cq values were transformed into a linear scale (2^-ΔCq^, where ΔCq = Cq – minimum Cq); for both BestKeeper and Silver’s method, mean Cq values were used. From these analyses we obtained the following measures: σ_Cq_ (Silver’s method), which represents the average standard deviation of the difference in cycle threshold values across all possible pairs of reference genes; M (geNorm), a measure of gene expression stability relative to all candidate reference genes; SD (BestKeeper), the raw standard deviation of Cq values relative to the software’s index measure; r (BestKeeper), the coefficient of correlation relative to the software’s index measure; and the Stability Value (NormFinder), representing expression variance in both the intragroup and intergroup analyses.

## Supplementary information

**Additional file 1: Figure S1.** Cq values for candidate reference genes in PBMCs and T-cells, stratified by age and stimulation status. Mean quantification cycle (Cq) values are presented for young (YN) and old (ON) unstimulated (−) and influenza A/Victoria/375 stimulated (+) donor PBMCs (A) and CD3+ T-cells (B). No significant differences (*p* > 0.20) were detected by Wilcoxon rank-sum test and data from a total of 10 donors is presented.

## Data Availability

The datasets used and/or analysed during the current study are available from the corresponding author on reasonable request.

## Abbreviations

ACTB: β-actin
CD3^+^: Cluster of differentiation 3
cDNA: Complementary deoxyribonucleic acid
CMV: Cytomegalovirus
Cq: Cycle of quantification
GAPDH: Glyercaldehyde-3-phostphate dehydrogenase
MIQE: The Minimum Information for Publication of Quantitative Real-Time PCR Experiments
O: Old participant
PBMCs: Peripheral blood mononuclear cells
PGK1: Phosphoglycerate kinase 1
qPCR: Quantitative polymerase chain reaction
rcf: Relative centrifugal force
RNA: Ribonucleic acid
RPL13a: Ribosomal protein, L13a
RPLP0: Ribosomal protein large, P0
RPS18: Ribosomal protein, S18
SD: Standard deviation
SDHA: Succinate dehydrogenase complex flavoprotein subunit A
UBE2D2: Ubiquitin-conjugating enzyme E2D2
Y: Young participant
σ_Cq_: Average standard deviation of ΔCt values across all possible pairs of reference genes
