# Supplementary material for: Reliable reference genes for the quantification of mRNA in human T-cells and PBMCs stimulated with live influenza virus
Source: BMC Immunol. 2020 Jan 31;21:4. doi: 10.1186/s12865-020-0334-8 (PMC6995044; doi:10.1186/s12865-020-0334-8)
Supplement: Supplementary file 1 — Additional file 1: Figure S1. Cq values for candidate reference genes in PBMCs and T-cells, stratified by age and stimulation status. Mean quantification cycle (Cq) values are presented for young (YN) and old (ON) unstimulated (−) and influenza A/Victoria/375 stimulated (+) donor PBMCs (A) and CD3+ T-cells (B). No significant differences (p > 0.20) were detected by Wilcoxon rank-sum test and data from a total of 10 donors is presented. [file 12865_2020_334_MOESM1_ESM.docx]

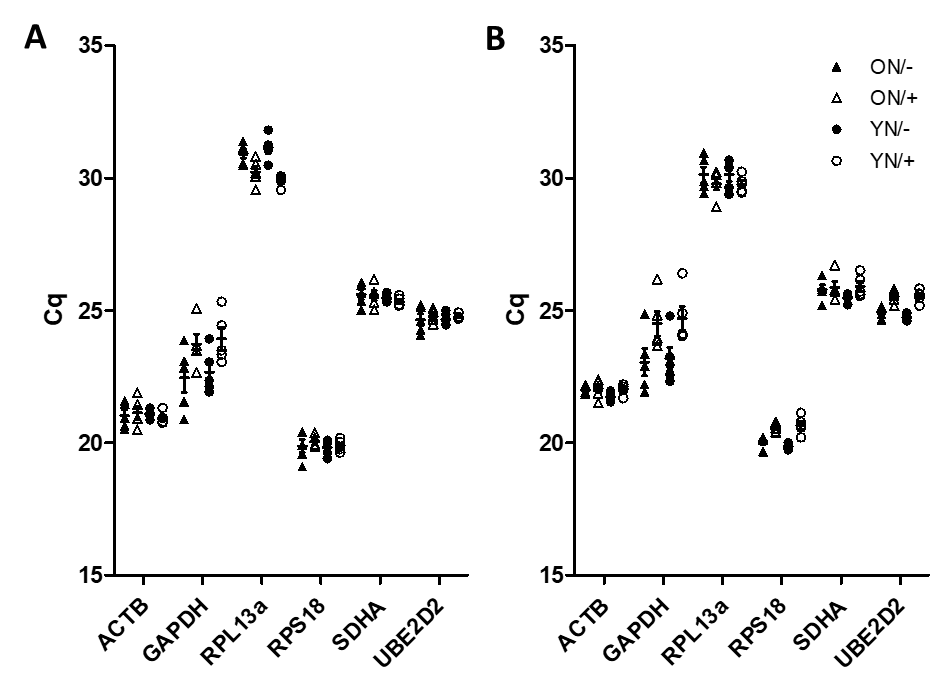


**Supplementary figure 1:** Cq values for candidate reference genes in PBMCs and T-cells, stratified by age and stimulation status. Mean quantification cycle (Cq) values are presented for young (YN) and old (ON) unstimulated (-) and influenza A/Victoria/375 stimulated (+) donor PBMCs (A) and CD3+ T-cells (B). No significant differences (p>0.20) were detected by Wilcoxon rank-sum test and data from a total of 10 donors is presented.
